# Reduced preference for social rewards in a novel tablet based task in young children with Autism Spectrum Disorders

**DOI:** 10.1038/s41598-017-03615-x

**Published:** 2017-06-12

**Authors:** Liliana Ruta, Francesca Isabella Famà, Giuseppe Massimo Bernava, Elisa Leonardi, Gennaro Tartarisco, Alessandra Falzone, Giovanni Pioggia, Bhismadev Chakrabarti

**Affiliations:** 1 Institute of Applied Sciences and Intelligent Systems, “Eduardo Caianiello” (ScienceApp) – National Research Council of Italy (CNR), via Torre Bianca, SNC, Istituto Marino, Pad. 4, 98164 Messina, Italy; 2 Department of Developmental Neuroscience, Stella Maris Scientific Institute, V.le del Tirreno 341, 56018 Calambrone, Pisa Italy; 3 Department of Cognitive Sciences, Psychology, Education and Cultural Studies (COSPECS), University of Messina, Via Concezione 6, 98122 Messina, Italy; 4 Centre for Integrative Neuroscience and Neurodynamics, School of Psychology and Clinical Language Sciences, University of Reading, Reading, RG6 6AL UK

## Abstract

Atypical responsivity to social rewards has been observed in young children with or at risk of Autism Spectrum Disorders (ASD). These observations contributed to the hypothesis of reduced social motivation in ASD. In the current study we develop a novel task to test social reward preference using a tablet computer (iPad), where two differently coloured buttons were associated with a social and a nonsocial rewarding image respectively. 63 young children, aged 14–68 months, with and without a diagnosis of ASD took part in the study. The experimental sessions were also recorded on video, using an in-built webcam on the tablet as well as an external camera. Children with ASD were found to show a reduced relative preference for social rewards, indexed by a lower proportion of touches for the button associated with the social reward image. Greater social preference as measured using the tablet-based task was associated with increased use of social communicative behaviour such as eye contact with the experimenter and social smile in response to the social reward image. These results are consistent with earlier findings from eye-tracking studies, and provide novel empirical insights into atypical social reward responsivity in ASD.

## Introduction

Autism Spectrum Disorders (ASD) is a complex of neurodevelopmental condition characterized by deficits in socio-communication skills alongside restricted range of interests and repetitive behaviour^1^.

One of the most commonly noted early signs of atypicalities in the social behavioural domains mentioned above is that of reduced responsiveness and/or attention to social signals. Developmentally, social signals such as social smiles and positive facial expressions are highly rewarding and are associated with social bonding in humans throughout lifespan^2–4^. Higher responsivity to social rewards is hypothesized to support communicative functions as well as social learning^5^. According to the Social Motivation theory of autism, reduced value of social stimuli in ASD can substantially contribute to the failure to preferentially attend to social stimuli^6^ and a subsequent loss of social learning opportunities^7–11^. A cumulative effect of this loss of social learning opportunities can adversely impact the typical development of the ‘social brain’^12, 13^.

Decreased social preference by 12 months of age is consistently reported in infants who are later diagnosed with ASD^14–20^. Using a free-view paradigm, greater preferential gaze for non-social stimuli such as geometric patterns in infants and toddlers has been found to be an early biomarker for a subset of children with ASD^21, 22^. At a neural level, atypical responses to social signals such as dynamic gaze shifts toward versus away from the infant at 6–10 months has been associated with an ASD diagnosis at 3 years of age^23^.

A large number of paradigms measuring social reward responsivity have used the passive viewing of social and nonsocial rewards^22, 24, 25^. Such paradigms typically measure the consummatory aspect of reward processing, related to the experience of ‘receiving’ a certain reward. Preference for a certain type of reward in such paradigms can manifest as greater looking time/ attention to social (such as people, faces and eyes) as opposed to non-social stimuli (i.e. food, money, objects). In such paradigms, mixed findings have been reported depending on the kind of stimulus used, the experimental procedures/design and methodological considerations^26^. While several studies using both static and dynamic visual scenes demonstrated that individuals with ASD consistently attend more to the non-social than social targets^8, 27–33^, other studies failed to find a significant group difference in viewing times for social when compared to non-social stimuli^34–41^.

A separate group of studies have rather focused on testing the “reward seeking” (related to the anticipatory aspect of reward processing), typically measured using deliberate choice or effort made to obtain the reward outcome.

Using event-related potentials (ERPs)in an incentive-delay task with social and nonsocial reward contingencies, atypical reward anticipation patterns have been noted in ASD and/or in relation to autism-related traits, with some studies reporting a specific deficit in the neural response to anticipation of social but not non-social rewards^42, 43^. Similar studies have observed a broader deficit for both social (positive facial expressions) and monetary reward contingencies^44^. Furthermore, fMRI studies suggested an atypical activation in the ‘reward related’ brain systems during anticipatory aspects of social reward processing in ASD^45–48^. Behavioural findings yield a mixed picture, with one study showing that 8–15 year old children with and without ASD use similar levels of effort to view pictures of faces^49^. In contrast, Dubey and colleagues found reduced preference for social stimuli in individuals with high autistic traits and ASD using a novel task that involved individuals making button presses to watch a social/nonsocial video clip (‘choose-a-movie’ paradigm)^49, 50^.

In our study, we developed a novel behavioural paradigm - developmentally appropriate for young children - to specifically target the seeking aspect of social motivation by testing the deliberate choice (measured using button presses) made by the child to see their preferred stimulus (either the image of a child smiling or a toy train). As such, this paradigm is similar in its approach to both the Ewing *et al*. and the Dubey *et al*. paradigms mentioned above, but is novel particularly because of the age group studied, as well as its child-friendly mode of administration (tablet computer). The design of this paradigm is based on one of the earliest studies that reported a reduced seeking of social (auditory) stimuli by young children with autism, using a toy with buttons that either led to a social or non-social sound^51^. One key innovation for the current study was to use the tablet computer, which children of this age in western European cultures are considerably familiar with, and makes the paradigm easy to administer (in the home setting) and scalable.

The task involved three processes that included a) an implicit categorizing of the presented stimuli coupled with b) the learning of the association between the categories and the different button colours, and c) making a motor response to the preferred button.

**Figure 1:**
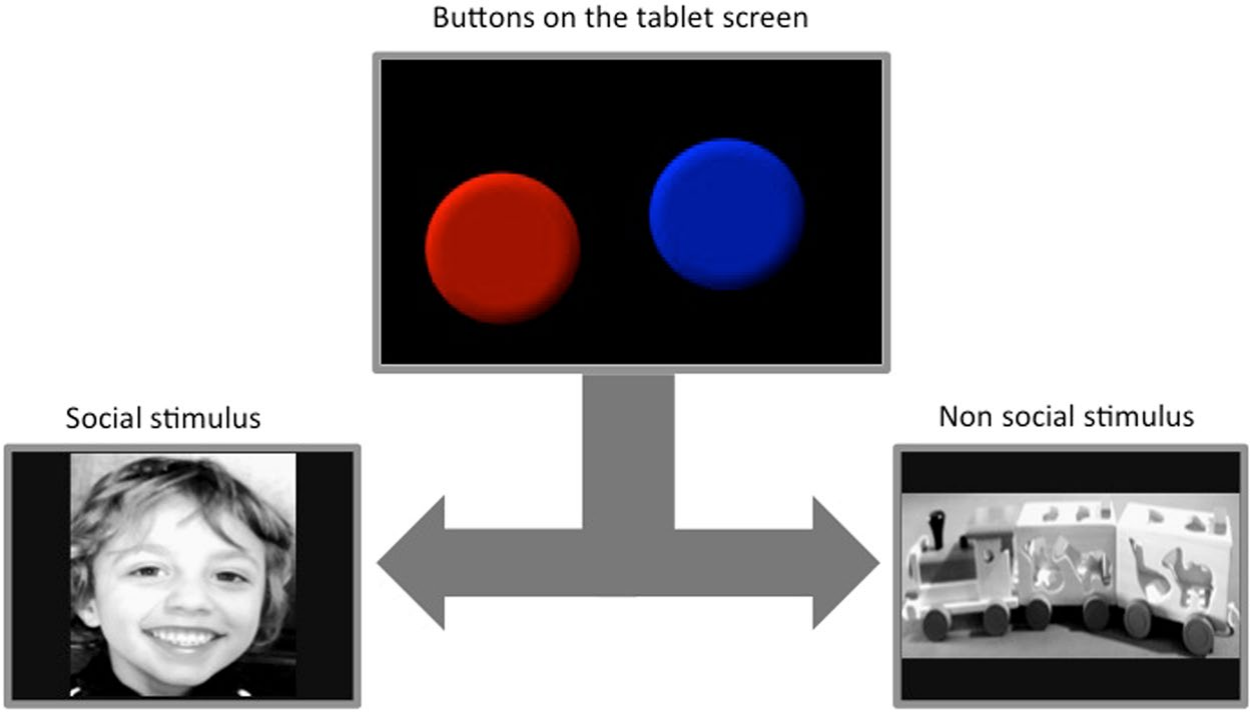
Schematic representation of a trial. The child would press one of the buttons on the screen in order to reveal either of the two pictures. These pictures would last for 3s, and then be replaced by the screen with the buttons after an inter-trial interval of 3s. Pictures used in this figure are representative, due to copyright restrictions.

In line with previous findings we predicted that young children with ASD will choose non social over social stimuli. In addition to measuring button presses, we further analyzed social communicative responses (such as smiles, gestures, pointing and vocalizations) to the stimuli. We hypothesised that typically developing children in comparison to children with ASD will exhibit a greater choice for social compared to non-social stimuli. We also predicted that the preference for social stimuli will be positively associated with the extent of social communicative responses.

## Results

Table 1 summarizes the demographic and clinical characteristics in the whole sample (n = 63). While the two groups were matched for age, there was a significant difference in gender and PDQ. Accordingly, all reported analyses are controlled for gender and PDQ scores.

**Table 1:** Demographic and clinical characteristics of the ASD and TD children (n = 58).

Measures	ASD (n = 21)	TD (n = 37)	p-values	95% CI	
	(Mean, SD)	(Mean, SD)		Lower	Upper
Age (months)	39.9 (11.5)	45.5 (10.7)	0.06	−11.7	0.350
Performace DQ^a^	92.38 (16.8)	136.1 (22.7)	<0.01	−55.1	−32.2
Handedness (Edinburgh)	0.32 (0.71)	0.60 (0.69)	0.16	−0.65	0.11
Male:Female	18:3	18:19	<0.01	—	—

ASD = Autism Spectrum Disorders; TD = Typically Developing children; CI = Confidence Interval of the Difference.

^a^Griffiths Mental Development Scales: Mean = 100, SD = 15.

The analysis of covariance showed a significant effect of group on social preference after controlling for gender and the PDQ scores (F(1, 53) = 10.92 p = 0.002, partial eta squared = 0.17), with the ASD group making lower proportion of button presses for social images (mean_ASD_= 0.37 [s.d. = 0.11] and mean_TD_ = 0.49 [s.d. = 0.12]). Due to a significantly higher number of males in the ASD compared to the control sample, this analysis was rerun in males only. This re-analysis revealed a similar pattern of results showing a lower preference for social images in ASD children F(1, 34) = 7.96 p = 0.008, (mean_ASD_ = 0.37 [s.d. = 0.1] mean_TD_ = 0.49 [s.d. = 0.1]).

**Figure 2:**
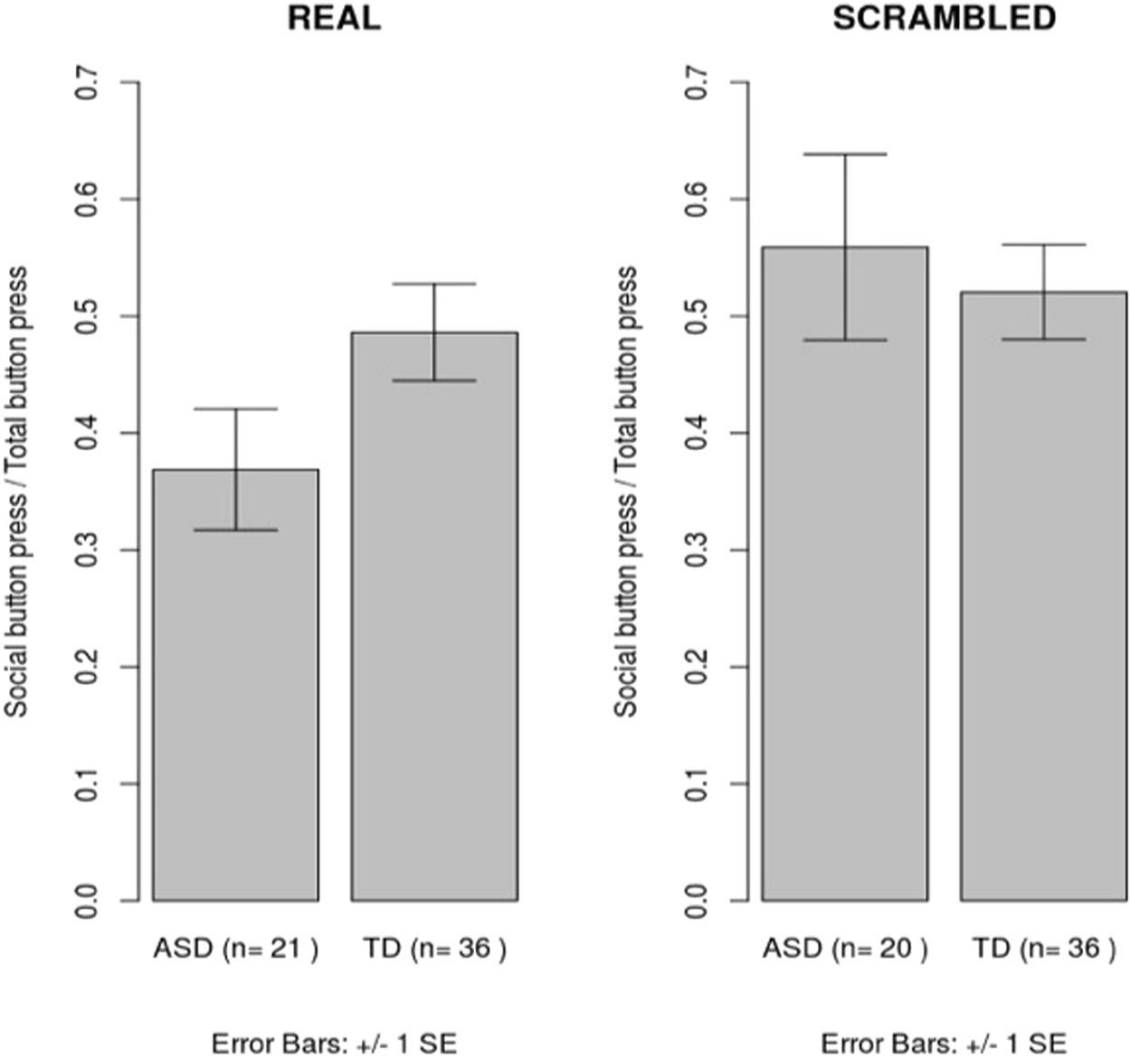
Group difference in relative preference for social stimuli for the real and scrambled image trials respectively.

No effect of group was noted for a similar analysis conducted on the data from the scrambled images (F (1, 53) = 0.08, p = 0.78, partial eta squared <0.01) (see Fig. 2

).

To explore the assumption that social versus non-social image preference was related to the reward value of the image, we analyzed the relationship between image preference and consequent child’s behaviours in the whole sample and in ASD and TD groups separately.

We found a significant positive correlation between the child’s relative preference for social image and the eye contact with the experimenter (*rho* = *0.39, p* < 0.001), as well as smiles, facial expressions and vocalizations to the screen in response to the social image (*rho* = 0.31, *p* < 0.001; *rho* = 0.20, *p* = 0.01 and *rho* = 0.22, *p* = 0.01 respectively). Consistently, we found a negative correlation between the relative preference for social images and pointing gestures to the image as well as vocalizations for non social images (*rho* = −0.20, p = 0.02 and *rho* = −0.34, *p* < 0.001 respectively). This pattern of correlation was seen both in ASD and TD groups separately, with minor group specific differences (see Table 2). Age and PDQ were not significantly correlated with preference for social image in ASD (*rho* = −0.2, p = 0.4 and *rho* = 0.4, p = 0.09) or TD children (*rho* = 0.09, p = 0.6 and *rho* = −0.08, *p* = 0.6 respectively).

**Table 2:** Correlations between social preference and child’s social behavior, after controlling for gender and PDQ, in the ASD and TD group.

SOCIAL IMAGE										
	e/c		Smile		Facial		Pointing		Vocal	
	ASD	TD	ASD	TD	ASD	TD	ASD	TD	ASD	TD
Socratio Spearman’s rho	0.43	0.22	0.40	0.18	0.26	0.09	0.49	−0.26	0.46	0.10
p-value	0.002^**^	0.035^*^	0.004^**^	0.06	0.048^*^	0.24	0.001^**^	0.014^*^	0.001^**^	0.19
**NON SOCIAL IMAGE**										
Socratio Spearman’s rho	0.14	−0.02	0.20	−0.17	0.26	−0.07	0.29	−0.35	−0.37	−0.09
p-value	0.19	0.42	0.11	0.08	0.048^*^	0.28	0.03^*^	0.001^**^	0.008^**^	0.22

Socratio = social/total button presses, e/c = number of eye contact with the experimenter, smile = number of smiles directed to the image, facial = number of facial expressions directed to the image, pointing = number of pointing gestures to the image, vocal = number of vocalizations during image presentation.

*p < 0.05, **p < 0.01, one-tailed.

## Discussion

Atypical preference for social stimuli has been widely reported in individuals with ASD and high autism-related traits. In this study, we used a novel tablet-based task to measure social preference in young children with and without ASD. We observed a reduced relative preference for social reward stimuli in toddlers with ASD compared to an age matched group of TD children. Further, we noted that this relative preference for social stimuli was positively correlated to multiple indices of ecologically valid social behaviour (e.g. eye contact, social smile, vocalization) exhibited by the child on seeing the social reward image.

A number of studies investigating social reward preference in this age group have used looking time as the metric of choice, as described systematically in a recent meta-analysis^26^. This meta-analysis confirmed a significantly reduced looking time (measured through fixation duration/ dwell time) to social stimuli in ASD, after accounting for potential publication biases. In contrast, the number of studies that measure anticipatory (/seeking) behaviour in relation to social rewards has been largely absent in this age group, possibly due to the limited motor repertoire. One study in slightly older children (6–8 year old) has observed a reduced electrocortical response during the anticipation of face stimuli in ASD^52^, while another study in an even older sample (8–15 year old) using an effort based task to view faces found no difference between children/adolescents with and without ASD^49^. Hence, this study addresses a niche that has been largely unexplored, through the use of an age-appropriate platform (a tablet computer, which toddlers are able to manipulate by simple touching of the screen), and stimuli (babies and toy trains). The use of gray scale images for the real stimuli, and a scrambled version for the control stimuli minimised the potential confounds due to differences in low-level stimulus properties. The choice of a single stimulus image (social and nonsocial respectively) ensured a developmentally appropriate cognitive demand of the task.

In support of our hypothesis, we observed a reduced preference for the button associated with the social image, in children with ASD. This result is consistent with the pattern of data noted in eye-tracking studies done in similar age groups that measure looking time to social and nonsocial stimuli^21, 22, 33, 53^. It is worth noting though that this pattern of reduced fixation to social stimuli has not always been noted in toddlers with or at risk for autism, and is likely to depend on stimulus type^24, 31, 54, 55^. In contrast to the eye-tracking studies using a passive viewing paradigm, we observed no preference for social rewards in the TD group - but a reduced preference for these stimuli in the ASD group. This observation is possibly due to the high salience of toy trains for this age group across the diagnostic divide. A second possibility is that the pattern of data is driven by increased social avoidance in the ASD group, rather than a reduced drive for seeking social stimuli. A third possible explanation is that the toy train represents a highly valued circumscribed interest in children with ASD, making them more likely to chose the train image.

Future experiments should aim to disambiguate between these three potential explanations. Crucially, we do not observe this reduced preference for social stimuli in ASD, when the images are replaced by their phase-scrambled versions, thus supporting our inference that the observed pattern of results is driven by the image content (social/nonsocial).

Children who showed a greater relative preference for the button associated with the social image, regardless of diagnostic status, also demonstrated higher social communicative behaviours (eye contact with the experimenter, social smiles, vocalization) in response to the social reward image. This positive correlation of the task-based measure of relative social preference with real-world behavioural responses to social rewards provides evidence of convergent validity for this new task.

Analysing the correlations separately for the ASD and TD groups reveals interesting insights. First, the pattern of correlations found in the whole sample were particularly consistent in the ASD group, especially in response to the social image. ASD children with higher relative preference for the social image exhibited more social behaviours such as smiles and eye contact with the experimenters, as well as pointing gestures and facial expressions. Puzzlingly however, these ASD children (who showed higher relative preference for the social image) also showed a higher number of pointing gestures and facial expressions to the non social reward image. In contrast, TD children with higher relative preference for the social image displayed fewer pointing gestures to screen at the social image. Although contrary to the expectations, it is possible that pointing gestures to the screen, exhibited by ASD children in response to both social and nonsocial images, represents a less sophisticated means of social communication, especially if not accompanied by gaze triangulation with the social partner.

These findings suggest that relative preference for social rewards exists in a continuum also within ASD. Children with ASD who showed a greater relative preference for the social image also demonstrated a greater number of of social communicative responses, particularly those indexed by social smiles and eye-contact.

As one of the early attempts to develop a novel task to measure social reward seeking using an age-appropriate platform and stimuli, this study needs to be replicated and developed further in larger, independent samples. Potential future directions for this study could involve modifications of the task to help overcome the limitations due to issues with maintaining of interest in the task in older children or reduced contingency understanding in younger children. One such modification involves replacing the static with dynamic stimuli, that are more interesting, and shown to be associated with larger group differences between ASD and TD children^54^. In our study, we chose static stimuli and their scrambled versions in order to provide a conservative control for potential confounds due to stimulus properties. Developing video stimuli that are matched for a large number of low-level stimulus properties pose a significant challenge. Another modification, arguably for older children, can involve building the contingency of each button with each category (e.g. social or nonsocial) rather than with a single stimulus image. Results from such a paradigm will be more generalisable across different stimuli and will minimise potential effects of habituation to a single stimulus. In view of the limitations of this preliminary empirical attempt to study social reward-seeking in toddlers with and without ASD using a mobile platform, we advise caution in generalizing our findings across all ASD children, or to reward seeking activities in day-to-day life.

The measure of choice in this paradigm is the relative preference for a button leading to a social image, similar to gaze bias metrics in preferential looking studies. As such, a lower value for this relative preference measure can be driven both by a true reduced preference for social rewards, as well as an increased preference for nonsocial rewards. The observed correlations of the relative preference for social images with social behaviours such as smiles and eye-contact provides some support for the former possibility.

In conclusion, we report the results of a new tablet-based social preference task in toddlers with and without ASD in this paper. We find a reduced relative preference for social reward images in toddlers with ASD when compared to TD children, as measured using the number of button presses made by the children to watch a social vs a nonsocial reward image. This result is consistent with several eye-tracking studies using a passive viewing paradigm, and provides a novel insight into the nature of social motivation impairments in ASD^56^.

## Methods

### Participants

Sixty-three children (25 ASD, [21 males and 4 females] and 38 typically developing (TD), [19 males and 19 females]), aged 14–68 months were enrolled in the study (see Table 1). This age range was governed primarily by the age-appropriateness of the task. Pilot results suggested that younger children did not understand the contingencies, while older children lost interest quickly. TD children were recruited through two mainstream nursery schools in Messina and Taormina (Sicily, Italy) and tested in a quiet room. ASD children were recruited as part of an ongoing research programme and tested at the clinical facilities within the National Research Council of Italy (CNR), Messina. All parents gave written informed consent, and the study protocol was approved by the Research Ethics Committee of the University of Messina. ASD diagnosis was made according to the DSM-5 criteria^1^ by an experienced multidisciplinary team including two child psychiatrists and 2 developmental psychologists. The Autism Diagnostic Observation Schedule - Second Edition (ADOS-2)^57^ was used as part of the diagnostic assessment. The Griffith’s Mental Development Scale (GMDS) was used to assess the Developmental Quotient^58^.

### Stimuli

The stimuli was created using Lua scripting language on top of an ObjectC framework (https://itunes.apple.com/app/stan/id914465120).

As shown in Figure 1, the stimuli consisted of two pictures, a toy train (non social) and a smiling face (social) - presented full screen on a 6.6 inches tablet computer (iPad). The stimuli were chosen among a group of five social and five non social images. Ten TD children aged three to five years old were showed all the images and asked for each image (i) if they liked it (Yes or No) and (ii) how much they liked it using a 3-point likert template with smiles. Furthermore, to control for non social stimuli that are of special interest to the ASD group, 10 parents of TD children and 10 parents of ASD children (aged 2 to 5 years) were asked to fill in a questionnaire on preferred toys for their child (listing them from the most to the least favourite). Toy trains appeared to be one of the most familiar and interesting toy for both ASD and TD children in the age range considered and was chosen as the non social reward image. We used one image of each type (social and nonsocial) in order to reduce the cognitive demands of the task, such that one button always predicted one image. Generalising across a category (e.g. one button predicting a category of ‘social’ images) is significantly more challenging for most children of this age, and particularly for children with learning delay. The two images were converted to grayscale and matched for image dimensions and luminosity. A scrambled version of these two pictures (8 × 8 pixel square scrambling operation based on double random phase encoding) was created and tested as control stimuli in order to ensure that any observed difference was not driven by low-level stimuli parameters. The real and scrambled images were presented in separate blocks, with the order of blocks being counterbalanced across participants. Each block consisted of eight trials of real or scrambled image trials. In each trial, two round buttons (55 mm diameter), one red and one blue, were presented on the screen, and were associated to the social and nonsocial image respectively. The button-image contingency was counterbalanced across participants. (Fig. 1).

### Procedure

All methods were carried out in accordance with the relevant guidelines and regulations specified by the Research Ethics committee. The child was sat on a comfortable chair in front of an iPad positioned on a support with a 45 degrees inclination to the table. The experimenter, sat in front to the child, started the training trial saying “[Child’s name], look!” and pressed one of the two buttons on the screen with the index finger contextually naming the button color. The aim of this training trial was to ensure that the child grasped the contingency between the button and the image. When the stimulus (either a slide or a guitar) appeared on the screen, the experimenter verbally labeled the object. The experimenter then pressed the other button naming the color and labelled the other image on the screen. Next the experimenter invited the child to do the same, saying “Now your turn!”. If the child did not touch the screen after the experimenter verbal prompt, the experimenter would physically prompt the child to touch the screen button once, leaving the last trial of the pilot session to be done independently by the child. If the child did not press the button independently during the last pilot trial, the test phase was not conducted assuming that the child had not adequate fine motor and/or cognitive skills to perform the task. If the child performed the pilot trial independently at least once after the modeling, the test trial was conducted without giving the child any further verbal prompt.

Each image was presented for 3 seconds immediately after the child’s touching the button on the tablet screen. A screensaver followed the image presentation for 3 seconds, following which the two buttons were presented again in a variable random position in the screen. The variable spatial positioning of the buttons was to ensure that the toddlers were not exhibiting their preference to a specific spatial location, but to a specific button.

The number and identity (social/nonsocial) of the button-presses were recorded. In addition, video footage of the session was recorded using both the tablet webcam (close-up of the child’s face) and a second external camera (recording the child’s face, tablet and experimenter’s face). Before commencing the main experiment, a separate block of 4 trials, identical to the test trial but using two different black and white images, was administered to familiarize the child with the task.

The examiner maintained a neutral face during the all test trial not to create biases in social engagement. Task understanding was coded from the video footage for all children by the experimenter according to the following criteria: a) active searching (through visual exploration of the screen and/or pointing gesture) of the corresponding button to the chosen image b) vocalizations related to the button color-image association. In a randomly chosen subgroup of n = 15 children from this sample, these behaviours were blind-coded by two independent coders, who were found to be mutually reliable (Cohen’s Kappa = 0.85). Furthermore, child behaviour directed to both the image on the screen and to the examiner - was analysed through video-coding. The following behaviours were manually coded from the video footage for all trials: eye-contact with the experimenter, smiles directed to the image, other facial expressions directed to the image, pointing gestures to the image, vocalizations during image presentation. Inter-rater reliability for coding each of these behaviours ranged from 0.85 to 1.

Five children (4 ASD and 1 TD aged 14 months) did not pass the pilot phase and were not administered the test phase, whilst performance developmental quotient (PDQ) subscore of the GMDS was not available for one TD child. Additionally, one child with ASD did not complete the control task (with scrambled images) and was excluded from the relevant analysis.

### Statistical analysis

Statistical analyses were conducted using IBM-SPSS Statistics 20 and R (http://www.r-project.org/).

Relative preference for social images was computed as a ratio ranging from 0 to 1 (number of social button touches/ total number of touches). Only the touches on the button were considered in the denominator. Random touches to the screen off the buttons were not computed. Between-group comparisons of relative preference for social images as well as correlation analyses with behavioural responses were examined in n = 58 children (n = 21 ASD children and n = 37 TD children). All the analyses were adjusted for PDQ and gender, since TD children had significantly higher PDQ compared to ASD children and the male to female ratio was not matched in the two groups.

One-tailed p values are presented for all the inferential statistics, in keeping with the directional nature of the hypotheses. Since behavioural variables showed significant deviation from normality, Spearman rank correlation (controlling for PDQ and gender) was used to assess the relationship between image preference and behavioural responses in the ASD and TD group separately.
